# Worldwide Meniere's disease research: A bibliometric analysis of the published literature between 2002 and 2021

**DOI:** 10.3389/fneur.2022.1030006

**Published:** 2022-10-12

**Authors:** Wujun Zou, Qian Li, Fei Peng, Dingqiang Huang

**Affiliations:** ^1^Department of Otorhinolaryngology Head and Neck Surgery, Chengdu Second People's Hospital, Chengdu, China; ^2^Department of Anesthesia, West China Hospital of Sichuan University, Chengdu, China

**Keywords:** Meniere's disease, CiteSpace, VOSviewer, bibliometric analysis, hotspot, trend

## Abstract

**Background:**

In recent years, there has been an increasing number of publications on Meniere's disease. However, there are no bibliometric research on Meniere's disease. The purpose of this study was to find the focus and trends of Meniere's disease research through bibliometric approach.

**Methods:**

Publications related to Meniere's disease in the Web of Science Core Collection (WOSCC) from 2002 to 2021 were collected. The bibliometric approach was used to estimate the searched data. Research foci of the studies were identified using VOSviewer and CiteSpace software.

**Results:**

A total of 1,987 articles meet the inclusion criteria and are included in the study. In the past 20 years, the number of Meniere's disease publications is gradually increasing, especially in the past 3 years. The country with the largest contribution to Meniere's disease research is the United States, followed by Europe and Japan. High-frequency keywords included Meniere's disease, endolymphaic hydrops, vertigo, meniere-disease, inner ear, dizziness, symptoms, hearing, diagnosis, and tentamicin. The analyses of keyword burst direction indicate that evoked myogenic potential, MRI, and committee are emerging research hotspots.

**Conclusion:**

This study provides an objective, systematic, and comprehensive analysis of Meniere's disease-related literature. In addition, we find a dramatic increase in studies in this field over the past 3 years. Evoked myogenic potentials and MRI may become the research hotspots of Meniere's disease in future. This study will help otolaryngologists, neurologists, and audiologists to clarify the research direction and potential hotspots of Meniere's disease and further help clinicians improve patients' prognosis.

## Introduction

Meniere's disease is characterized by recurrent episodes of spontaneous, usually rotational vertigo, sensorineural hearing loss, tinnitus, and a feeling of fullness or pressure in the affected ear. It is a condition that frequently lasts for decades. It is usually unilateral but may be bilateral. Meniere's disease is most common between the ages of 30 and 60 years, although younger people may be affected (1–3). Acute episodes can occur in clusters of about 6–11 years, although remission may last many months or even years. In addition, many people with Meniere's disease often have reduced daily activities to avoid triggering the disease. However, limiting daily activities may delay psychological and neurophysiological recovery, prolong the onset of the disease, and increase distress (4, 5).

Despite the availability of various interventions, there is uncertainty surrounding their relative efficacy, thus making it difficult to select the appropriate treatments for Meniere's disease (6, 7). Most treatments provide only temporary relief of symptoms, not the psychiatric symptoms associated with Meniere's disease, such as anxiety, irritability, and depression. Thus, it is imperative to find effective treatments for Meniere's disease.

Bibliometric analysis uses mathematical and statistical methods to analyze basic information on included literature, such as countries/regions, research collaborations, journals, institutions, and authors, and to identify research dynamics in selected areas (8, 9). Bibliometric analysis can be used to predict potential research hotspots by showing changes in research directions at different time points, and it has been used in many research fields (10). Compared with systematic reviews and meta-analysis, bibliometric analysis can comprehensively analyze the current status of research fields and more accurately predict development trends (11). CiteSpace is a Java-based scientific mapping software, and VOSviewer helps to build maps based on web data, both of which were used for analyzing included literature (12). Both CiteSpace and VOSviewer have been used in various fields, such as physical education and healthy urban planning (13, 14).

Bibliometric techniques and visual analysis have not been used to summarize Meniere's disease research. At the same time, there is no research to explore the hotspot of Meniere's disease. In this study, we aim to investigate the research status and hotspots of Meniere's disease in the past 20 years through quantitative analysis. Furthermore, we build a network of authors, institutions, and countries among the researchers. These analyses can bring new perspectives to otolaryngologists and neurologists.

## Materials and methods

### Data source and search strategy

**Figure 1:**
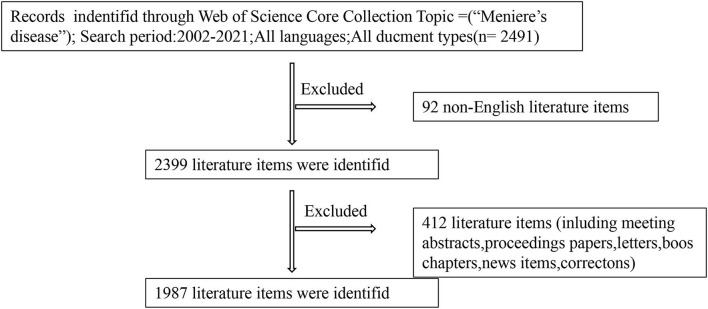
Frame flow diagram of search strategy.

We carefully searched the Web of Science database for articles about Meniere's disease from 2002 to 2021 and included original articles and review articles. Topic = (Meniere's disease) AND Language = English was the only retrieval strategy. On 1 July 2022, we completed the literature search and data collection. Since there were no exclusion criteria, the search results might include some non-relevant articles. The detail of search procedure is exhibited in Figure 1. Two researchers analyzed the data separately, and if there was a disagreement, a third researcher would be asked to solve the problem. The information includes title, abstract, keywords, author, institution, country, journal, references, and citations.

### Bibliometric analysis

First, the included data were converted to texts, which included basic information such as co-cited articles, keywords, countries, institutions, journals, authors, and network characteristics of keyword burst. Second, the texts were imported into the analysis software including. Finally, the results are presented in tables or figures. The Journal Citation Report 2021 included the H-index, impact factor, and category quartiles. Among them, the H-index is an important indicator for evaluating the scientific impact of journals or articles (15, 16).

To explore the research hotspots of Meniere's disease, we analyzed the included articles by CiteSpace software. The metrics analyzed were publishers, co-cited articles, and most relevant keywords. Different nodes on the network visualization map represent different analyzed objects. The larger nodes indicate more frequent occurrences. Moreover, we analyzed the centrality of the included studies by CiteSpace software, where the more important nodes are represented *via* higher centrality (17). We presented the evolution of research fields and collaborations between different institutions by creating scientific knowledge networks through VOSviewer software followed by predicting potential research hotspots.

VOSviewer software was used to analyze the co-occurrence of keywords. The results were presented as a density map with different color clusters showing the co-occurrence frequency of the keywords which has the potential to predict underlying trends.

## Results

### Annual outputs and growth trends

**Figure 2:**
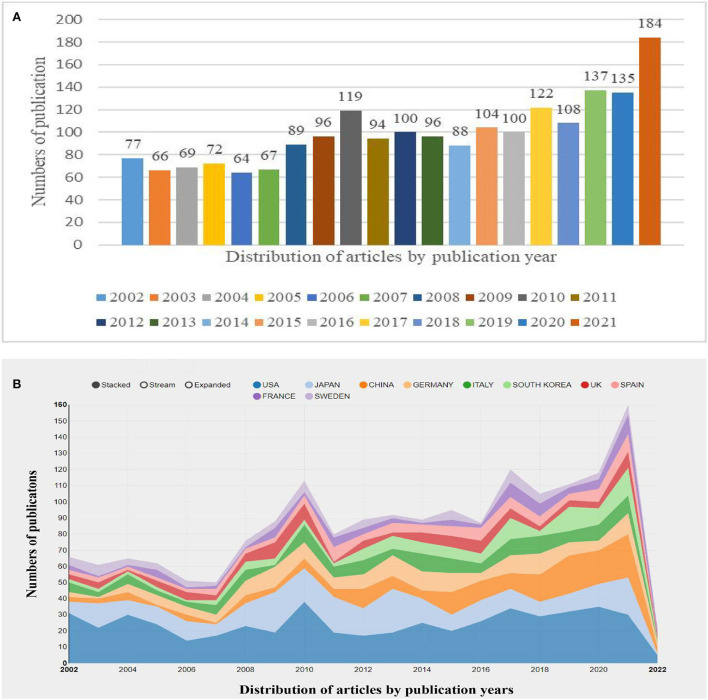
Trends in the number of publications **(A)** and the top 10 countries/regions **(B)** on Meniere's disease research from 2002 to 2021.

Overall, there has been an upward trend in original research on Meniere's disease over the past 20 years, especially in the last 3 years. In the past two decades, researchers have published a total of 1987 studies (Figure 2A). In Meniere's disease research, the United States published the most research results (Figure 2B). In the past 3 years, the number of articles published has increased significantly. In the past decade, research on Meniere's disease has peaked, almost twice as much as in the previous decade.

### Distribution of Countries/Regions and Institutions

**Figure 3:**
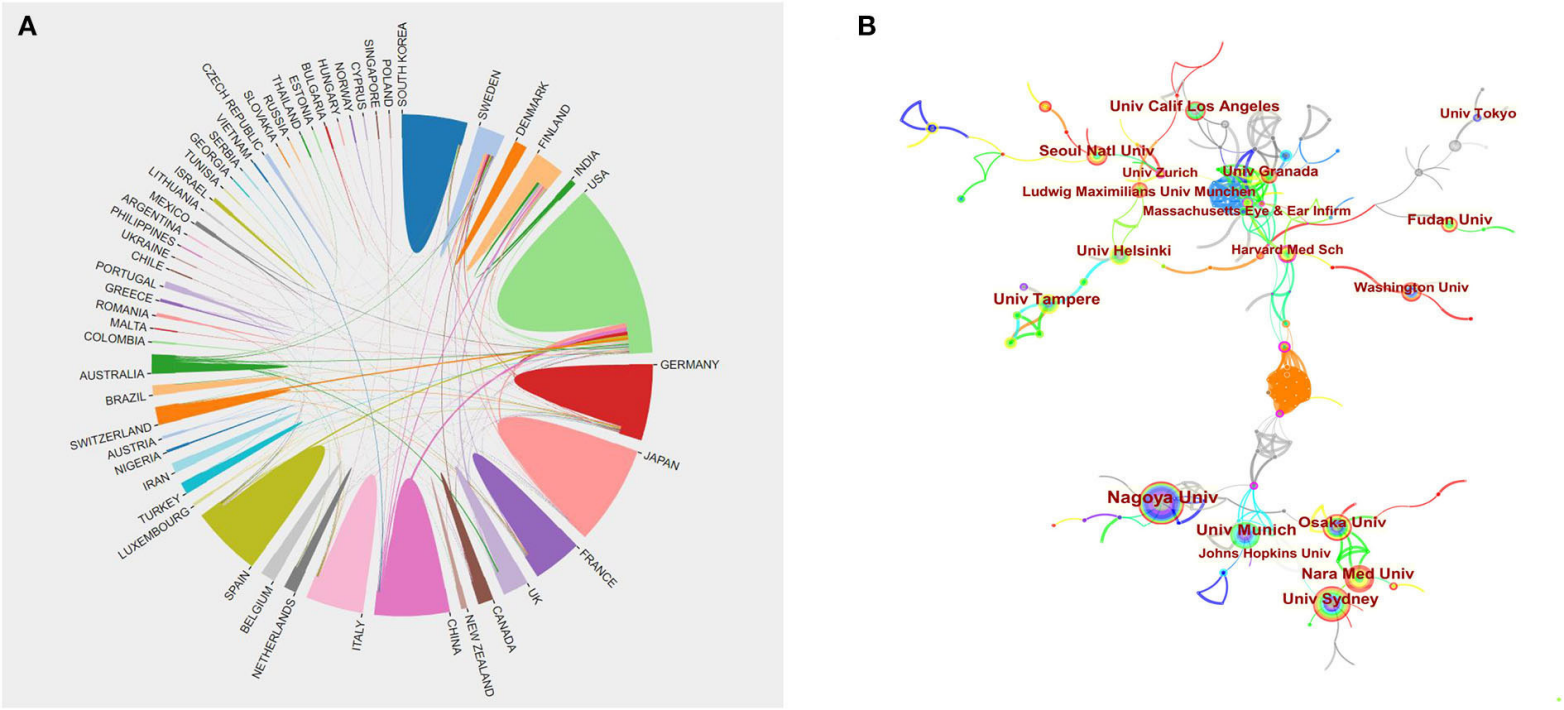
Cooperation of countries/regions **(A)** and institutions **(B)** contributed to publications on Meniere's disease research from 2002 to 2021.

A network of national collaborations between studies on Meniere's disease is shown in Figure 3A. Table 1 shows the top ten contributing countries. The country with the most published research is the United States (513), followed by Japan (296), Germany (170), China (145), and Italy (142). The United States accounted for about 30% of the total number of published studies. The centrality score is a metric for evaluating the importance of network nodes. The centrality analysis of this study confirms that the United States (0.72) is at the top of the network, followed by Spain (0.35) and France (0.31). Higher centrality means stronger cooperation. The low density of the national cooperation network graph indicates that the research institutions are relatively independent and need to strengthen further cooperation.

**Table 1:** Ranking of top 10 most published countries in Meniere's disease research from 2002 to 2021.

**Rank**	**Articles counts**	**Centrality**	**Country**
1	513	0.72	USA
2	296	0.00	Japan
3	170	0.04	Germany
4	145	0.04	China
5	142	0.08	Italy
6	120	0.00	South Korea
7	101	0.35	Spain
8	87	0.08	UK
9	86	0.31	France
10	85	0.19	Sweden

The network of institutional collaborations between studies on Meniere's disease is shown in Figure 3B. The top ten institutions include University of Munich (0.02), University of California (0.01), Nagoya University (0.09), Tampere University (0.03), and Harvard University (0.03) (Table 2). The highest centrality is Massachusetts Eye and Ear Infirmary (0.17). The low centrality of all institutions indicates less inter-agency cooperation and further emphasizes the importance of cooperation.

**Table 2:** Ranking of top 10 institutions for collaboration in Meniere's disease research from 2002 to 2021.

**Rank**	**Articles counts**	**Centrality**	**Institutions**	**Country**
1	75	0.02	University of Munich	Germany
2	62	0.01	University of California	USA
3	61	0.09	Nagoya University	Japan
4	54	0.00	UDICE	France
5	47	0.03	Harvard University	USA
6	47	0.03	Tampere University	Finland
7	43	0.09	University of Sydney	Australia
8	38	0.17	Massachusetts Eye and Ear Infirmary	USA
9	38	0.05	Osaka University	Japan
10	35	0.05	University of Navarra	Spain

### Contributions of authors

**Figure 4:**
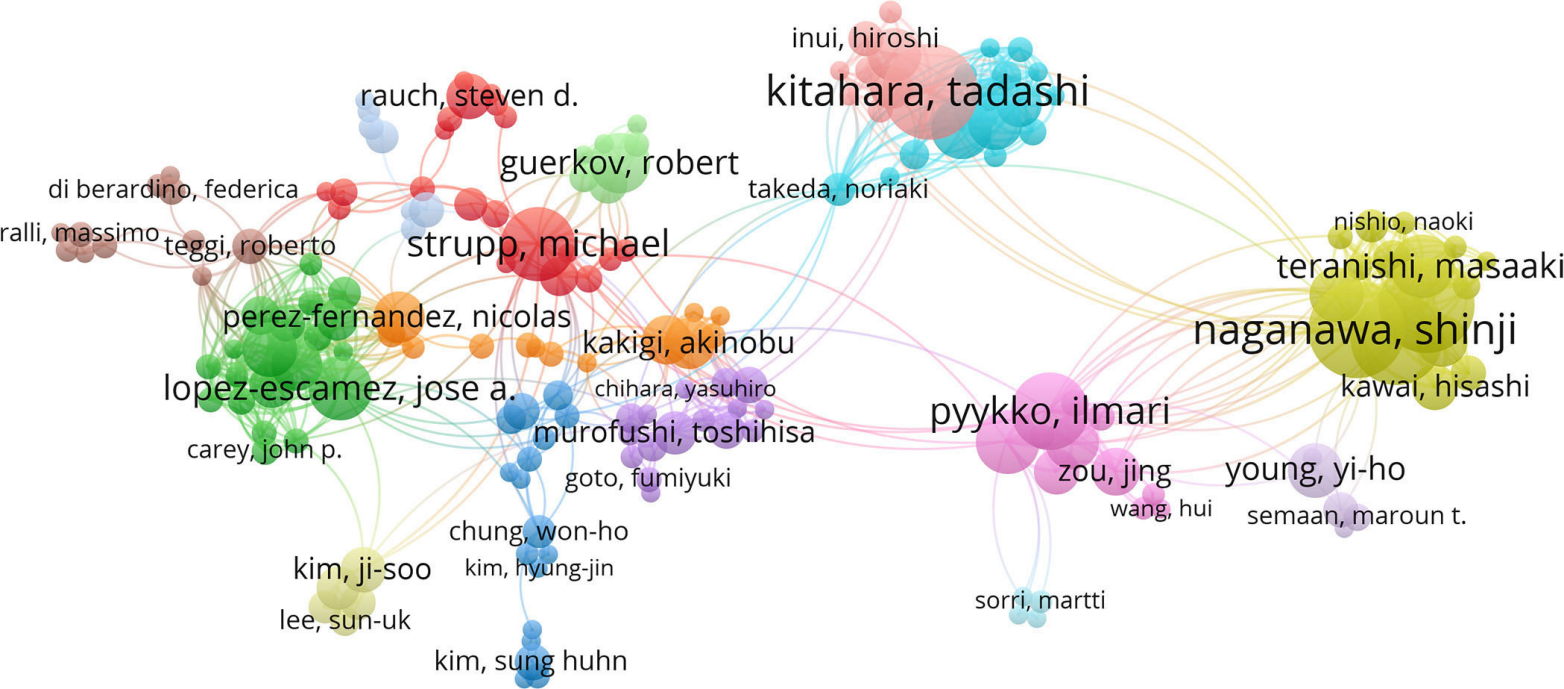
Joint mapping of productive authors in Meniere's disease research from 2002 to 2021.

VOSviewer was used for visualizing authors (at least five articles and 200 citations) (Figure 4). In the past 20 years, 266 authors have published ≥ five studies on Meniere's disease. Each circle represents a researcher. Some overlapping authors may not be presented. Closed circles represent researchers with closed working relationship.

Table 3 shows the top ten researchers with the most published articles. The most published author is Kitahara T (53 articles, 659 citations), followed by Naganawa S (51 articles, 2,153 citations). In terms of centrality, Nakashima T and Strupp M tied for first place (0.08). The centrality of investigators working on Meniere's disease was all below 0.1, which emphasized that Meniere's disease investigators were less cooperative and needed to strengthen their cooperation with each other.

**Table 3:** Ranking of top 10 most published authors in Meniere's disease research from 2002 to 2021.

**Rank**	**Author**	**Articles Counts**	**Centrality**	**Total Citations**	**Average Citations**	**H-index**
1	Kitahara T	53	0.02	659	12.43	13
2	Naganawa S	51	0.06	2,153	42.22	25
3	Sone M	51	0.07	1,858	36.43	23
4	Nakashima T	50	0.00	2,136	42.72	25
5	Pyykko I	43	0.08	836	19.44	13
6	Lopez-escamez JA	41	0.06	1,720	41.95	22
7	Strupp M	35	0.08	2,292	65.49	23
8	Kentala E	33	0.02	517	15.67	12
9	Teranishi M	30	0.02	1169	38.97	18
10	Gurkov R	28	0.00	760	27.14	17

### Journal analyses

Table 4 presents the characteristics of the top ten active journals. Most of the journal's publishers are located in the United States and Europe. The top three journals that publish Meniere's disease research are Otology and Neurotology, Acta Oto-Laryngologica, and Laryngoscope. Otology and Neurotology publishes the most articles on Meniere's disease. Moreover, Otology and Neurotology had the highest average number of citations (29.16) and the highest H-index (35). Journal Citation Report quartile Q1 included Otolaryngology-Head and Neck Surgery, Q2 contained European Archives of Oto-Rhino-Laryngology and Frontiers In Neurology, Q3 contained Otology and Neurotology, Laryngoscope, Journal of Laryngology And Otology, Auris Nasus Larynx, Annals of Otology Rhinology And Laryngology and Audiology And Neuro-Otology, and Q4 contained Acta Oto-Laryngologica in.

**Table 4:** Ranking of top 10 journals for number of published articles on Meniere's disease research from 2002 to 2021.

**Rank**	**Journal**	**Articles counts**	**Country**	**Journal citation reports (2021)**	**Impact factor (2021)**	**Total cites**	**Average number of citations**	**H-index**
1	Otology and Neurotology	263	USA	Q3	2.619	5,177	19.68	35
2	Acta Oto-Laryngologica	224	Norway	Q4	1.698	3,159	14.10	26
3	Laryngoscope	104	USA	Q3	2.970	3,033	29.16	30
4	European Archives of Oto-Rhino-Laryngology	91	Germany	Q2	3.236	1,204	13.23	19
5	Otolaryngology-Head And Neck Surgery	62	USA	Q1	5.591	1,257	20.27	20
6	Journal of Laryngology And Otology	56	UK	Q3	2.187	603	10.77	14
7	Frontiers In Neurology	55	Switzerland	Q2	4.086	488	8.87	12
8	Auris Nasus Larynx	51	Netherlands	Q3	2.119	559	10.96	12
9	Annals of Otology Rhinology And Laryngology	43	USA	Q3	1.973	548	12.74	14
10	Audiology And Neuro-Otology	42	USA	Q3	2.213	563	13.40	14

### Cluster analysis of keyword co-occurrence related to research hotspots

**Figure 5:**
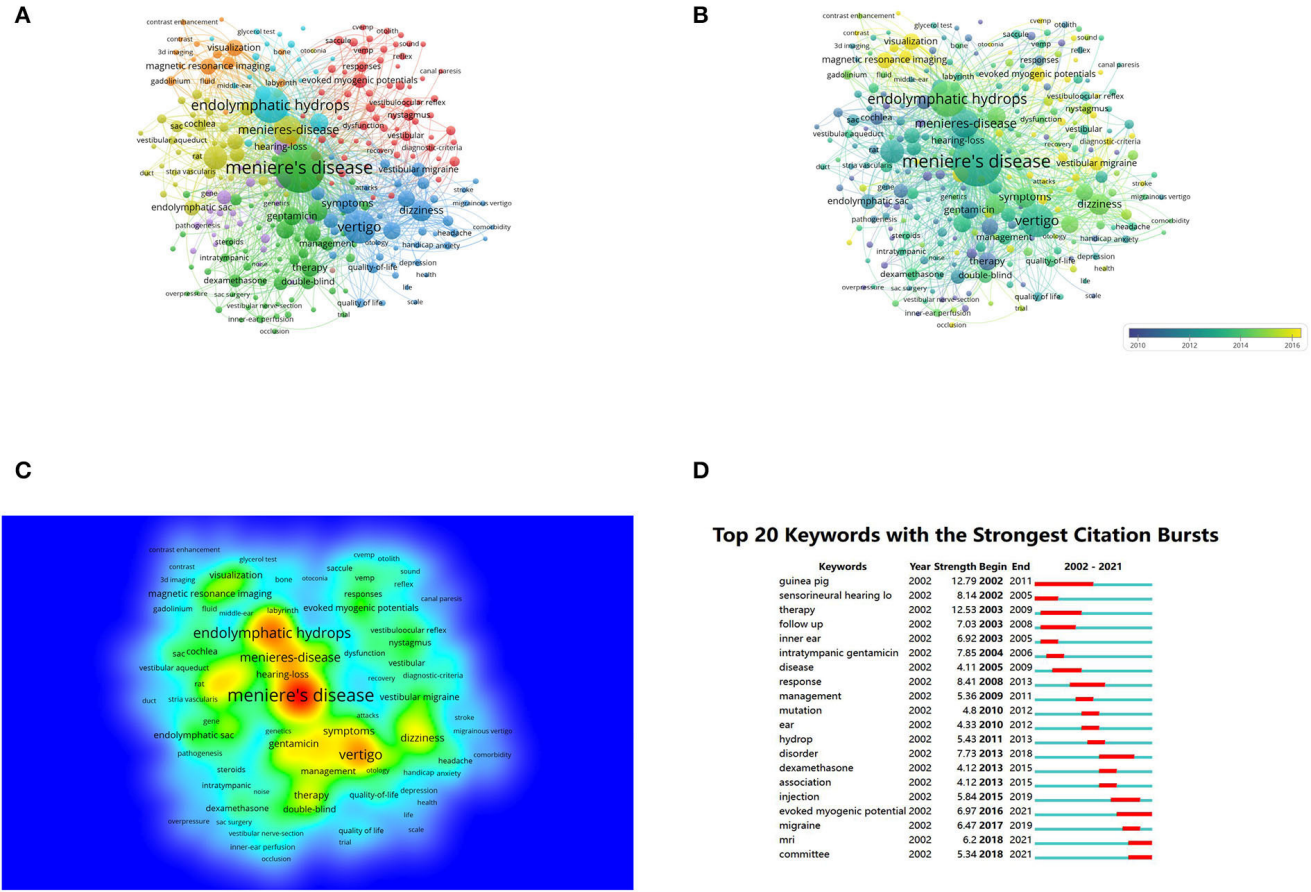
Co-occurrence analysis of global research on Meniere's disease based on the WoSCC database from 2002 to 2021. **(A)** Mapping of keywords in the research field. **(B)** Distribution of keywords according to the chronological order of appearance. **(C)** Distribution of keywords according to the mean frequency of appearance. **(D)** Keywords with the strongest citation bursts in Meniere's disease research.

The titles and keywords of the 1,987 literature included in this study were analyzed *via* VOSviewer software. Figure 5A depicts that the map, which was divided into five clusters, contained only 252 keywords (more than 20 times) and 4,979 keywords totally. The frequency keywords were meniere's disease (998), endolymphaic hydrops (518), vertigo (453), meniere-disease (327), inner-ear (223), dizziness (185), symbols (165), hearing (154), diagnosis (136), and tentamicin (135). Terms with similar research subjects were combined under the same category, with five main clusters: clinical characteristics, mechanisms, diagnosis, management, and pathophysiology of Meniere's disease.

Figure 5B shows the distribution of keywords that appear in order. The order of keywords is determined by the color of the labels. In the first decade, most research focused on mechanisms and pathophysiology, and recent research trends suggest that diagnosis may become a research hotspot.

Moreover, Figure 5C shows that the frequency of occurrence of different keywords is represented by the density of the graph. The higher the density, the warmer the color “closer to yellow.” Research hotspots in this field mostly appear in areas with high density.

### Detection of keyword bursts

By analyzing 1,987 articles retrieved from the WoSCC database, we identified keyword outbreaks from 2002 to 2021 (Figure 5D). A straight line represents the timeline, consisting of blue and (or) red. The red part illustrates the burst period in which length represents the starting and ending year and time span. To focus on research trends in Meniere's disease, we identified keywords with strongest burst. Between 2002 and 2021, guinea pig (12.79) was top one, followed by therapy (12.53), response (8.41), sensorineural hearing loss (8.14), and intratympanic gentamicin (7.85). The analyses of keyword burst direction indicated that evoked myogenic potential (2016–2021), MRI (2018–2021), and committee (2018–2021) were emerging research hotspots.

### Analyses of co-cited references

**Figure 6:**
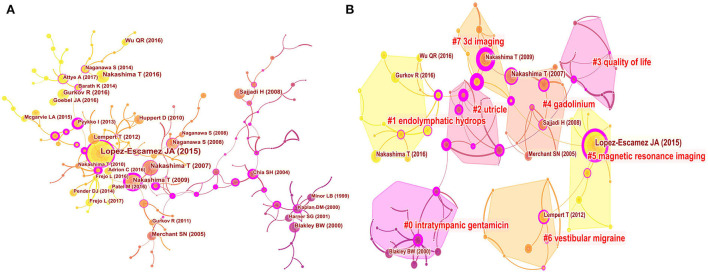
Co-cited references map **(A)** and clustered network map of co-cited references **(B)** on Meniere's disease research from 2002 to 2021.

In this study, a co-citation analysis was performed on 25,639 cited references from 1987 articles, and a clustering network graph was obtained based on the analysis of the results. The visual network of the co-cited articles consists of 184 nodes and 204 links (Figure 6A). Cited articles are represented by nodes. The diameter of the node represents the total number of cited articles. Links between nodes indicate how often the citation was cited. The purple ring around the node can be used to connect the stages of the growth of a field. The red around the node represents the referenced “explosive growth”.

**Figure 7:**
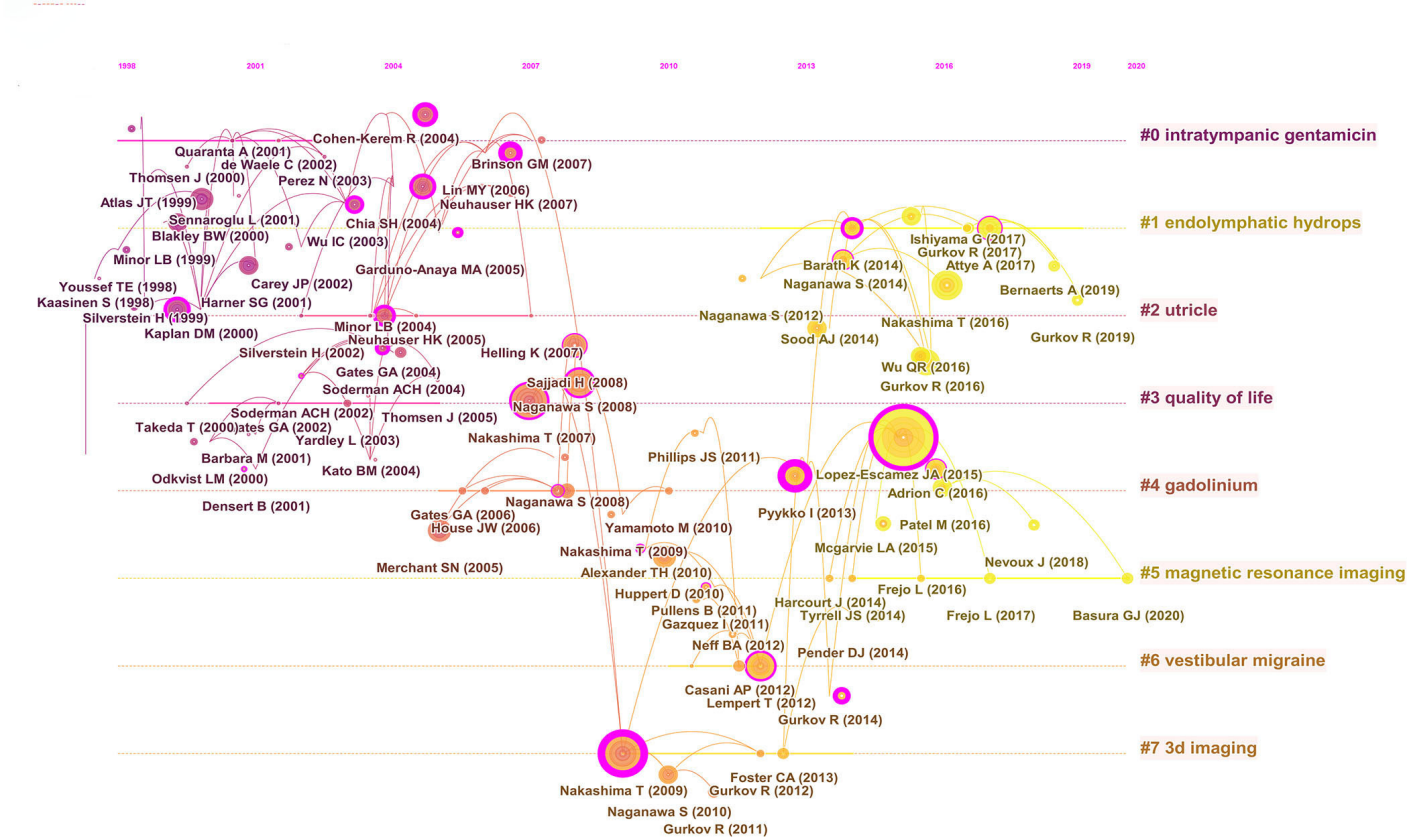
Timeline view of co-cited clusters with cluster labels.

Moreover, the research hotspots of Meniere's disease can be found by ranking the co-cited articles generated in the co-citation network. There were eight major clusters (Figure 6B). Figure 7 presents a timeline perspective of the cluster plot, supporting the discovery of new research hotspots in Meniere's disease. Table 5 lists the top 10 co-cited articles. The study published by Lopez-Escamez JA (18) in the Journal of Vestibular Research-Equilibrium and Orientation was the most cited (577 citations), followed by the research published by Nakashima T (19) in Laryngoscope (343 citations) and the article published by Merchant SN (20) in Otology and Neurotology (324 citations).

**Table 5:** Top 10 most co-cited references on Meniere's disease research from 2002 to 2021.

**Rank**	**Title**	**First Author**	**Year**	**Journal**	**Cited Frequency**
1	Diagnostic criteria for Meniere's disease	Lopez-Escamez JA	2015	Journal of Vestibular Research-Equilibrium & Orientation	577
2	Visualization of endolymphatic hydrops in patients with Meniere's disease	Nakashima T	2007	Laryngoscope	343
3	Pathophysiology of Meniere's syndrome: Are symptoms caused by endolymphatic hydrops?	Merchant SN	2005	Otology & Neurotology	324
4	Meniere's disease	Sajjadi H	2008	Lancet	255
5	Epidemiology of vertigo	Neuhauser HK	2007	Current Opinion In Neurology	237
6	Migraine and Meniere's disease - Is there a link?	Radtke A	2002	Neurology	221
7	Intratympanic dexamethasone for sudden sensorineural hearing loss after failure of systemic therapy	Haynes DS	2007	Laryngoscope	199
8	Bilateral vestibulopathy: Diagnostic criteria Consensus document of the Classification Committee of the Barany Society	Strupp M	2017	Journal of Vestibular Research-Equilibrium and Orientation	193
9	Causative factors and epidemiology of bilateral vestibulopathy in 255 patients	Zingler VC	2007	Annals of Neurology	174
10	Epidemiology of vertigo, migraine and vestibular migraine	Lempert T	2009	Journal of Neurology	156

**Figure 8:**
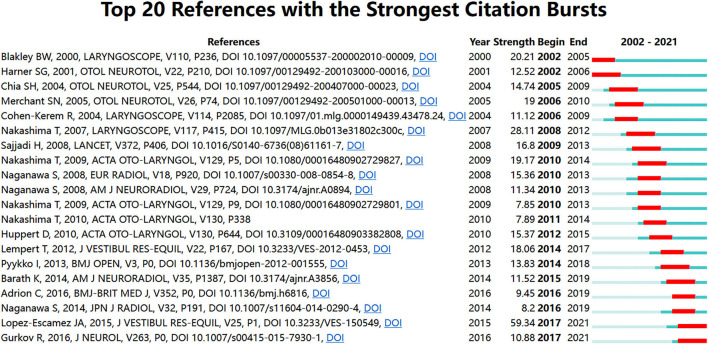
Top 20 references with the strongest citation bursts.

**Figure 9:**
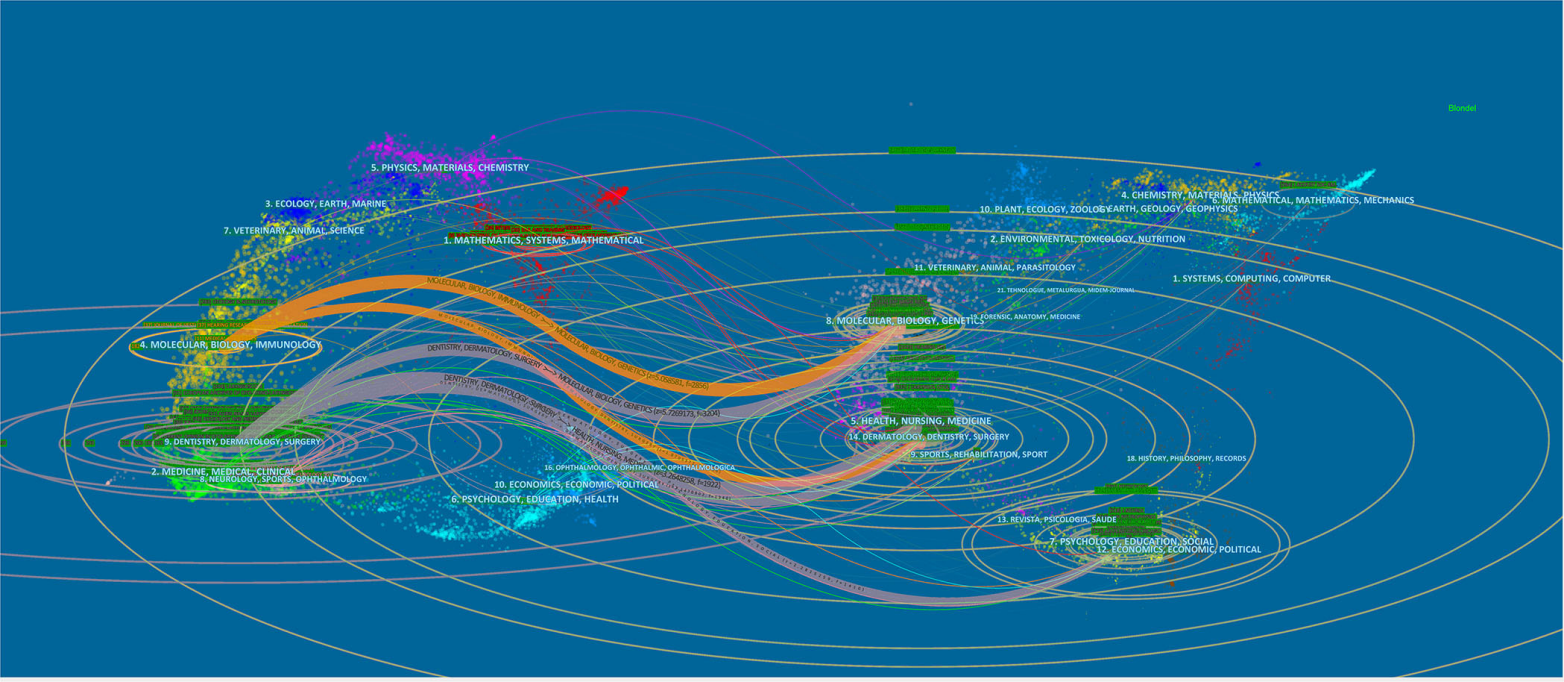
Dual-map overlay of journals on Meniere's disease.

Figure 8 shows the top 20 most cited references. Most of the highly cited references were from ENT or neurology publishers. This suggests that Meniere's disease belongs to the intersection of otolaryngology and neurology. The citing journal is on the left, and the cited journal is on the right. There are five main reference paths in Figure 9: three gray paths and two orange paths. The orange path means that studies published in Molecular/Biology/Immunology journals are cited in Molecular/Biology/Immunology/Dermatology/Dentistry/Surgery journals. Studies published in Dermatology/Dentistry/Surgery journals are cited for studies in Molecular/Biology/Genetic, Health/Nursing/Medicine, and psychology/Education/Social journals, as shown by the gray route.

## Discussion

Meniere's disease is an inner ear disease characterized by endolymphatic hydrops (21). Meniere's disease is often misdiagnosed due to the difficulties to directly see the endolymphatic hydrops and defects with auxiliary testing equipment. Therefore, it is imperative to summarize Meniere's disease research in recent years. Interestingly, this is the only study to analyze the research status of Meniere's disease by quantitative and qualitative bibliometrics, which includes 5,000 articles retrieved from WoSCC. The results confirmed that the total number of publications on Meniere's disease worldwide was gradually increasing over the past 7 years, which suggested the importance in otolaryngology and neurology. The number of articles had increased dramatically over the past 3 years. On the one hand, it may benefit from the improvement of assistive technology and clinical treatment. On the other hand, it may be related to experimental technology and research investment. The United States accounted for 30% of the articles on Meniere's disease. Furthermore, the publishers are mainly located in the United States and Europe. This result confirms that there are good medical conditions in developed countries, especially in European and American countries. At the same time, this result also reflects the urgency of strengthening global cooperation to find effective treatments for Meniere's disease. In addition, China, Japan, and Germany have also published many articles, but these countries lack cooperation with each other, so these countries have relatively low centrality. We suggest that countries should strengthen cooperation to jointly promote the progress of Meniere's disease research which is eventually beneficial for patients. In terms of Meniere's disease research, the top 10 institutions are from USA, Europe, and Japan, which confirm that research in developed countries is far ahead of relatively developing countries. University of Sydney, Massachusetts Eye and Ear Infirmary, and Nagoya University have the strongest collaborations with other institutions. Moreover, it suggests that there should be greater cooperation between institutions with less interaction.

Kitahara T has published the most articles in the field of Meniere's disease. Kitahara T's research focuses on the study of Meniere's disease, mainly including clinical research, diagnostic technology, international consensus, and diagnostic and therapeutic strategies. In addition, Naganawa S, Sone M, Nakashima T, and Pyykko I are the five most published scholars in the past two decades. Naganawa S, Nakashima T, Sone M, and Strupp M were the top four authors with a high H-index. Nonetheless, scholars who have made outstanding contributions to Meniere's disease research are mostly from developed countries, such as the United States, Europe, and Japan. Therefore, enhanced communication and collaboration among researchers around the world will facilitate the development of Meniere's disease research.

Meniere's disease-related articles are mostly published in leading journals in the field of ENT and neurology, including Otology & Neurotology, Acta Oto-Laryngologica, Laryngoscope, European Archives of Oto-Rhino-Laryngology, Otolaryngology-Head and Neck Surgery, Journal of Laryngology And Otology, Frontiers In Neurology, Auris Nasus Larynx, Annals of Otology Rhinology And Laryngology, and Audiology And Neuro-Otology. These journals are recognized as leading journals within the specialty, greatly contributing to the development of ENT and neurology. This trend suggests that otolaryngologists and neurologists are dedicated to the disease together. Judging from the references cited, researchers are currently focusing more on the clinical management of Meniere's disease. It is worth mentioning that the first most cited reference is the article “Diagnostic Criteria for Meniere's Disease” published by Lopez-Escamez JA (18), which proposes normative diagnostic criteria for Meniere's disease. This is a milestone in the clinical treatment and management of Meniere's disease. At the same time, a dual-map (Figure 8) overlay also confirms that Meniere's disease's research focus has begun to transform from basic research to clinical research. The core of basic research includes Molecular, Biology, and Immunology. Such translation brings great benefits to Meniere's disease patients.

Judging by the frequency of keywords, the epidemiology, pathophysiology, and treatment of Meniere's disease remain as the focus of research. Burst keywords indicate cutting-edge and emerging trends in scientific research. Three frontiers of Meniere's disease research were identified: evoked myogenic potential (2016–2021), MRI (2018–2021), and committee (2018–2021). Meniere's disease is an extremely distressing disease, one of the reasons is that it is easy to be misdiagnosed due to the lack of clear diagnostic criteria. Thanks to the existence of the Committee on Hearing and Equilibrium of the American Academy of Otolaryngology (AAO-CHE), the clinical guidelines of Meniere's disease were clarified, which greatly improved the diagnosis and treatment of Meniere's disease (22). Vestibular evoked myogenic potentials (VEMPs) are short-latency vestibular reflexes primarily driven by otoliths and are often evoked by air-conducted sound (ACS), bone-conducted vibration (BCV), or electrical vestibular stimulation (23, 24), although vestibular function tests alone have limited diagnostic value for Meniere's disease. However, the diagnosis of Meniere's disease still needs a comprehensive evaluation with vestibular function examination (25). In 2007, scholars confirmed the existence of endolymphatic hydrops in Meniere's disease patients using MRI imaging with intratympanic gadolinium (19, 26). Although MRI is not the gold standard for the diagnosis of Meniere's disease, it still contributes to the development of Meniere's disease (27).

The treatment of Meniere's disease has been a challenge for neurologists and otolaryngologists for a long time. The pathogenesis of Meniere's disease is mainly related to idiopathic endolymphatic hydrops in the cochlear duct and vestibular organs (28). Current treatments for Meniere's disease mainly include conservative drugs such as diuretics and betahistine, intratympanic steroids, endolymphatic sac surgery or intratympanic gentamicin, and destructive surgery (29, 30). The onset of clinical symptoms of Meniere's disease is often random, and there may be periods of remission lasting months to years. For the reason, the misdiagnosis, which results in poor outcomes, is common (31). In particular, vestibular migraine (VM) and Meniere's disease overlap in clinical symptoms in patients without hearing loss, and these disorders may co-occur, with some studies estimating a 35% incidence of VM in Meniere's disease patients (32).

We observe that endolymphatic hydrops, genes, and immunology are the focus of research in Meniere's disease. Endolymphatic hydrops is an area of lymphatic fluid accumulation in the inner ear that occupies the perilymphatic space, mostly in the cochlear duct and saccule, and occasionally in the utricle and semicircular canal (21). At present, endolymphatic hydrops can be visualized by injecting gadolinium contrast agent, which increases the diagnostic rate of Meniere's disease (33). Scholars have confirmed that the degree of endolymphatic hydrops is related to the stage of Meniere's disease, low-frequency hearing threshold, EchoG, and VEMP asymmetry ratio (34, 35). Meniere's disease is mostly attributed to endolymphatic hydrops in the inner ear. However, some evidence supports genetic contribution to Meniere's disease. The incidence of Meniere's disease varies in different ethnic backgrounds and geographical regions. In different regions, the incidence of Meniere's disease was 43/100.000 (in Finland), 56/10.000 (in UK), 200/100.000 (in USA), and 17-34.5/100.000 (in Japan) (31, 36, 37). Genetically, variant forms of Meniere's disease include monogenic forms in isolated families and polygenic forms in most familial and sporadic cases. Mutated genes in sporadic cases include GJB2, USH1G, SLC26A4, ESRRB, CLDN14, NTN4, and NOX3 (38). In familial Meniere disease, mutated genes include FAM136A, DTNA, PRKCB, COCH, DPT, SEMA3D, STRC, HMX2, TMEM55B, OTOG, and LSAMP (39). Moreover, multiple rare missense variants in the OTOG gene are related to 33% familial Meniere disease (40). The role of immune mechanisms in the development of MD is also a hot topic. The possible immune mechanisms of Meniere's disease include type I allergy, autoimmunity, circulating immune complexes, and immune genetics. The inner ear might participate in immune responses as autoantigens, including cellular and humoral immunity (41, 42). This allows neurologists and otolaryngologists to obtain more accurate pathogenesis, which can help improve the diagnosis and treatment of Meniere's disease. This will also make precision therapy and targeted therapy possible.

### Limitations

Certain limitations should be acknowledged. First, the constant updating of the database may lead to discrepancies between the number of articles retrieved and those included. Second, although the data for bibliometric analysis mainly come from the WoSCC database, some articles from other databases may be missed (43, 44). In addition, the absence of books, chapters, letters, and non-English literature may contribute to the bias of our analysis. Finally, different countries invest differently in Meniere's disease researches, which may be a potential source of bias. Nonetheless, this study presents the status and trends of Meniere's disease research through bibliometric analysis. Visual analysis of published literature helps researchers quickly understand hotspots and trends in Meniere's research, providing a basis for finding new research directions.

## Conclusion

The number of publications in the field of Meniere's disease was steadily increasing over the past two decades. Research on Meniere's disease may become a hotspot in neurology and otolaryngology in future. There are far more articles published in the journals of otolaryngology and neurology than other journals. The United States contributes ~30% of Meniere's disease publications, which greatly improved the treatment status of Meniere's disease. Meanwhile, China is the only developing country in the top 10 countries that contributed mostly in the relative fields. Therefore, it is urgent to strengthen the collaboration between different research teams. Further research into Meniere's disease will benefit a wide range of patients. Endolymphatic hydrops, pathophysiology, and therapy are the current research hotspots in the field of Meniere's disease.

## Data availability statement

The original contributions presented in the study are included in the article/supplementary material, further inquiries can be directed to the corresponding author.

## Author contributions

WZ and DH conceived the study. WZ, QL, and FP collected the data. WZ and FP re-examined the data and analyzed the data. WZ wrote the manuscript. FP reviewed and revised the manuscript. All authors contributed to the article and approved the submitted version.

## Conflict of interest

The authors declare that the research was conducted in the absence of any commercial or financial relationships that could be construed as a potential conflict of interest.

## Publisher's note

All claims expressed in this article are solely those of the authors and do not necessarily represent those of their affiliated organizations, or those of the publisher, the editors and the reviewers. Any product that may be evaluated in this article, or claim that may be made by its manufacturer, is not guaranteed or endorsed by the publisher.
